# The Healthy Heart

**Published:** 1921-03-19

**Authors:** 


### THE HEALTHY HEART.

In a lecture delivered at the Institute of Hygiene on March 9, Dr. Strickland Goodall said it is probably correct to say that there is no organ in the body about which people are so concerned as the heart, and this for two reasons : First, because the heart is associated in the mind of the general public with sudden death; and second, because from time immemorial the heart has been associated with the emotions. In this connection he has analysed all the cases of sudden death within his experience, and found that as a rule it is emotion that kills, and not hard work. A broken heart is a physiological possibility, and he has worked out the actual mechanism whereby the heart may be "broken." He has known an animal "break" its heart from joy.

The obvious thing for a cardiologist to do is to study the healthy heart, for this is the standard from which the diseased heart is going to depart. In order to get the accurate size and form of this vital organ Dr. Goodall has *x*-rayed a large number of healthy students of the Middlesex Hospital, and, after allowing for distortion due to magnification, he has made up a hypothetical heart, and come to the conclusion that the average transverse diameter of the heart in a man under thirty years of age is about 5¼ inches.

As one gets older the heart tends to become wider, for both the form and size of the heart vary with age. In a child the heart is more conical in shape and is relatively larger than in the adult. Moreover, a child's heart differs in position from that of an adult, and is usually irregular in action, speeding up during inspiration and slowing down during expiration. Sometimes the slowing-down process is so marked that the heart almost stops beating; that is actually the sign of a healthy heart in a child. The heart sounds in a child differ from those of an adult, and they closely resemble the sounds from an adult heart the muscles of which are diseased. A child's perfectly healthy heart is often mistaken for a diseased heart because it appears to be larger, is irregular, and sounds different from the heart of an adult.

In sedentary workers who take little exercise the heart becomes extremely soft and flabby. Such people are very liable to palpitation and other signs of heart disease. It is hearts of this type that are very liable to be pressed upon by the distended stomach, due to over-feeding, and that have made the fortunes of German Spas.

With regard to the capacity of the heart for work, he estimates this at 30,000 foot-pounds in nine minutes, and the heart should return to its normal condition in fifteen minutes. That would be a first-class heart, however. A definite experiment on a man running to catch a train has shown that before starting his heart was doing 152 foot-pounds of work a minute; after finishing his run it was doing 360 foot-pounds a minute. An experiment on a man going upstairs showed an increase in the work of the heart of 112 foot-pounds in walking up, and 152 foot-pounds in running up the stairs. The result of getting into a temper, taken from actual observation, showed an increase of the heart's work of 72 foot-pounds; therefore, keep your tempers.

Every heart has a certain amount of reserve energy, but we must try to realise to what extent we call upon this reserve. In all these various acts we not only put a strain on the heart at the time, but its total work for a considerable period afterwards is increased. To keep the heart healthy you must have sufficient exercise, adequate rest, and adequate nutrition; and to get this nutrition you must not only have a good blood supply, but an adequate nerve supply.

